# A comparative analysis of terrestrial arthropod assemblages from a relict forest unveils historical extinctions and colonization differences between two oceanic islands

**DOI:** 10.1371/journal.pone.0195492

**Published:** 2018-04-25

**Authors:** Mário Boieiro, Thomas J. Matthews, Carla Rego, Luis Crespo, Carlos A. S. Aguiar, Pedro Cardoso, François Rigal, Isamberto Silva, Fernando Pereira, Paulo A. V. Borges, Artur R. M. Serrano

**Affiliations:** 1 Centre for Ecology, Evolution and Environmental Changes (cE3c)/Azorean Biodiversity Group & University of Azores, Departamento de Ciências e Engenharia do Ambiente, Angra do Heroísmo, Azores, Portugal; 2 School of Geography, Earth and Environmental Sciences (GEES), The University of Birmingham, Birmingham, United Kingdom; 3 Biodiversity Research Institute (IRBio), Department of Evolutionary Biology, Ecology and Environmental Sciences, University of Barcelona, Barcelona, Spain; 4 Centre for Ecology, Evolution and Environmental Changes (cE3c) & Faculty of Sciences, University of Lisbon, Lisbon, Portugal; 5 Finnish Museum of Natural History, University of Helsinki, Helsinki, Finland; 6 CNRS-Université de Pau et des Pays de l’Adour, Institut des Sciences Analytiques et de Physico-Chimie pour l'Environnement et les Materiaux IPREM, MIRA, UMR, BP, Pau Cedex, France; 7 Madeira Natural Park, Funchal, Madeira, Portugal; Universite Paris-Sud, FRANCE

## Abstract

During the last few centuries oceanic island biodiversity has been drastically modified by human-mediated activities. These changes have led to the increased homogenization of island biota and to a high number of extinctions lending support to the recognition of oceanic islands as major threatspots worldwide. Here, we investigate the impact of habitat changes on the spider and ground beetle assemblages of the native forests of Madeira (Madeira archipelago) and Terceira (Azores archipelago) and evaluate its effects on the relative contribution of rare endemics and introduced species to island biodiversity patterns. We found that the native laurel forest of Madeira supported higher species richness of spiders and ground beetles compared with Terceira, including a much larger proportion of indigenous species, particularly endemics. In Terceira, introduced species are well-represented in both terrestrial arthropod taxa and seem to thrive in native forests as shown by the analysis of species abundance distributions (SAD) and occupancy frequency distributions (OFD). Low abundance range-restricted species in Terceira are mostly introduced species dispersing from neighbouring man-made habitats while in Madeira a large number of true rare endemic species can still be found in the native laurel forest. Further, our comparative analysis shows striking differences in species richness and composition that are due to the geographical and geological particularities of the two islands, but also seem to reflect the differences in the severity of human-mediated impacts between them. The high proportion of introduced species, the virtual absence of rare native species and the finding that the SADs and OFDs of introduced species match the pattern of native species in Terceira suggest the role of man as an important driver of species diversity in oceanic islands and add evidence for an extensive and severe human-induced species loss in the native forests of Terceira.

## Introduction

Relative to their area, islands make a disproportionately large contribution to global biodiversity but have long been severely impacted by human intervention leading several authors to consider that the present biodiversity crisis is particularly acute in island ecosystems [1–4]. Over the last few centuries most extinctions have taken place on oceanic islands, mainly as a consequence of direct and indirect human actions, particularly overhunting, habitat destruction, habitat fragmentation and species introductions [3–6]. Further, simply by considering the severe habitat devastation reported for many islands and the acknowledged vulnerability of island endemic invertebrates, we have become aware that major extinctions on oceanic island ecosystems often remain unnoticed [7, 8]. The restricted range size and small populations of many island endemic invertebrate species clearly highlight how these species may be particularly susceptible to extinction [7, 9, 10].

In recent years there has been a growing interest in the assessment of invertebrate extinction. For example, relevant information of high extinction levels on island ecosystems has been put forward for molluscs, a group of invertebrates where the presence of a shell is crucial to evaluate changes in community composition across time [3, 4, 8, 11, 12]. In contrast, the number of documented extinctions of terrestrial arthropods is small, even though this species-rich group of invertebrates has undoubtedly been the most severely affected by human driven extinctions over the last few centuries [7, 13–15]. The assessment of species vulnerability to extinction has relied mostly on the study of extinction risk indicators (e.g. life-history traits, rarity, population decline and fragmentation) due to the lack of detailed population viability analysis data [16]. In terrestrial arthropods, the characterization of rarity and conservation status of species is hampered by the poor information on species distributions and abundances over time, and sensitivity to ecosystem disturbance [17]. Consequently, the assessment of terrestrial arthropod rarity has mostly depended on the analysis of recent data on species abundances and distributions [18, 19].

The joint analysis of species abundance distributions (SADs) and of occupancy frequency distributions (OFDs) is a very useful tool for identifying patterns of commonness and rarity of species in communities, allowing inferences on the processes underlying community assembly, and providing valuable information for scientists and conservation managers [20, 21]. However, no direct association should be established *a priori* between the observed low-abundance range-restricted species and their vulnerability to extinction because this group of species may include tourists and poorly-sampled species along with the truly rare ones [18, 22, 23]. Many pseudo-rare species are classified as rare as a consequence of spatial, phenological and methodological edge effects [23, 24], and may account for a substantial fraction of the range-restricted species group. Unless properly identified, pseudo-rare species may blur the interpretation of commonness-rarity patterns in ecological communities [18].

In this study, we compare the assemblages of epigean spiders and ground beetles from the native forests of two oceanic islands–Terceira (Azores archipelago) and Madeira (Madeira archipelago)—using standardized data from a broad sampling program. We specifically selected these islands since both host important areas of a relic forest—the Laurisilva—that is restricted to just three Macaronesian archipelagos, but also due to the contrasting conservation status of their native forests. In Terceira, the native forest was severely destroyed and fragmented by anthropogenic activities since human colonization during the 15^th^ century, and presently occupies a small fraction of the island area [25, 26]. In contrast, the extent of forest destruction in Madeira has been less severe and some large pristine forest patches are still present [27, 28]. Thus, the comparative analysis of the spider and ground beetle assemblages of the native forests from both islands may provide insights about the consequences of habitat destruction on the composition and structure of invertebrate communities. In addition to both study islands hosting several of the remaining areas of Laurisilva, they share other characteristics in common, such as having climatic and edaphic affinities, whilst differing in others, such as geographic, geological and historical features [26, 28, 29](Table 1; see also the study area section). Madeira is older, larger, higher and closer to mainland (and to paleoislands), thus hosting higher species and habitat diversity [26, 28, 30, 31]. Nevertheless, Madeira has a much larger human population than Terceira, receives nearly a million visitors each year (over ten times that of Terceira) and has for long been a strategic stopover on the transatlantic trade routes. Despite the enduring and severe threats to Madeira biodiversity, the Laurisilva survived mainly due to the complex orography of the island.

**Table 1:** Main characteristics of Terceira and Madeira islands and of their native forests. Geographic, geological and ecological characteristics of the two study islands with information on their native forests. Data on tree species composition and on the actual and potential distribution of native forest in Madeira and Terceira were obtained from several references ([26, 27, 28, 32, 33] and references therein).

	Terceira	Madeira
**Area (km**^**2**^**)**	402	741
**Altitude (m)**	1023	1862
**Age (MY)**	3.5	5
**Ontogenetic stage**	Immature–lower geomorphological complexity	Mature–higher geomorphological complexity
**Distance to nearest island/mainland (km)**	37/1520	20/660
**Native forest area remaining/potential (km**^**2**^**)**	23/402	150/600
**Temperature in native forest areas (°C) (minimum—maximum)**	13.1–15.2	10.7–16.2
**Humidity in native forest areas (%) (minimum—maximum)**	94.7–98.9	87.7–97.4
**Precipitation in native forest areas (mm) (average)**	2497	1753
**Common tree species genera in native forest**	*Erica*, *Ilex*, *Juniperus*, *Laurus*, *Vaccinium*	*Clethra*, *Ilex*, *Laurus*, *Morella*, *Ocotea*, *Persea*, *Vaccinium*
**Human colonization**	15^th^ century	15^th^ century
**Human population**	56,062	256,014

This study aimed to (i) examine and compare SADs and OFDs of epigean spiders and ground beetles in Terceira and Madeira islands, (ii) evaluate the contribution of introduced and rare species to SAD and OFD profiles, and (iii) assess how species compositional differences of spider and ground beetle assemblages relate with the legacy of human disturbance on the native forests of both study islands. Taking into consideration the higher disturbance in Terceira native forests, we expect to find a higher number of introduced species and a lower proportion of endemics in this island than in Madeira. Further, based on previous works that found an association between departures from log-normality and the effects of disturbance on community structure (see a recent review by [21] and the references therein), we hypothesize that the logseries may best fit the SADs from the more disturbed forests of Terceira, while the SADs from Madeira Laurisilva will be better modelled by the lognormal distribution. Finally, we predict that introduced species will be restricted to low-abundance and low-occupancy classes in Madeira Laurisilva, but in Terceira they will present a wider class distribution in both SADs and OFDs.

## Materials and methods

### Study area

The study was undertaken in the native forests of Madeira and Terceira islands. These two oceanic islands belong to different volcanic archipelagos (Madeira and Azores) that lie in the North Atlantic: Madeira is located between latitudes 32°-33° N and longitudes 16°-17° W, while the Azores range between 37°-40° N and 25°-31° W. The Madeiran archipelago comprises three island groups—Madeira, Porto Santo and the surrounding islets and Desertas—but only the main island has native laurel forest (Laurisilva). The Azores is a relatively recent archipelago, comprising nine islands and several additional islets. Presently, only seven out of the nine Azorean islands still have native forest fragments [25, 34, 35]. These native forest fragments can be dominated by *Erica azorica* or *Juniperus brevifolia*, or present a larger number of co-dominant tree species including the Azorean laurel (*Laurus azorica*). For simplicity, we will refer to them here as Laurisilva fragments. The Laurisilva is considered a relict subtropical forest where sclerophyllous laurel tree species from the genera *Laurus*, *Ocotea* and *Persea* are dominant, together with a few other tree species (e.g. *Clethra arborea*, *Ilex* spp., *Morella faya* and *Vaccinium* spp.). This kind of forest occupied a vast area in southwestern Europe and northwestern Africa during the Tertiary, but became extinct in most of its range following the progressive climatic change that culminated with the Pleistocene glaciations, being now restricted to the Macaronesian archipelagos. In both the Azorean and Madeiran islands, the Laurisilva covered most of the land surface before human settlement [27]. However, the Azorean Laurisilva suffered a drastic reduction of over 97% and became extinct in two islands (Corvo and Graciosa) during the mid 20^th^ century [25, 35]. The percentage of native forest cover in the remaining seven Azorean islands is low, ranging between 0.1–10.9%, and in most cases the remaining native forest is also highly fragmented and disturbed [26, 35]. In Terceira, only five native forest fragments survived the severe and generalised human impact, occupying less than 6% of the island surface (i.e. 23 km^2^) [26, 35]. Even so, a few of these forest fragments still harbour a substantial number of endemic species and were considered priority areas for biodiversity conservation in the Azores [19, 34, 36]. These forest fragments are now included in a recently created protected area–the Terceira Island Natural Park.

In Madeira, an extensive area of Laurisilva was also destroyed for timber, fuel and field clearing for agricultural use during the last few centuries, but the complex topography of the island allowed its survival and it now occupies nearly 20% of the island surface (i.e. 150 km^2^). During the second half of the 20^th^ century, the implementation of a multidisciplinary program to protect and recover natural plant cover coupled with the creation of a protected area—the Madeira Natural Park—were two major landmarks in allowing the conservation of Madeira Laurisilva. More recently, due to its outstanding natural value and the pristine condition of many forest areas, Madeira Laurisilva was included in the World Heritage List and the same reasoning has led to the inclusion of the Madeira archipelago within a global biodiversity hotspot [37].

The native forests of Terceira and Madeira share some characteristics since some of the dominant trees are congeners that have speciated in each archipelago and they also show floristic affinities and similarities in climatic and edaphic conditions [38, 39]. Still, there are some abiotic and biotic differences between the two forests (for example in species richness, composition, forest stature, soil humidity) that result from their different altitudinal ranges, historical factors, and the geographic location and geological age of each study island (Table 1; [26, 28, 29]).

### Study species and fieldwork sampling

Two groups of epigean terrestrial arthropods—spiders (Araneae) and ground beetles (Coleoptera, Carabidae)—were selected as targets of this study since they are ecologically important in most terrestrial ecosystems and their diversity, taxonomy and ecology is well known in the Azores and Madeira following comprehensive studies carried out in both archipelagos during the last two decades [30, 31, 40]. Spiders and ground beetles are two species-rich groups of terrestrial arthropod predators that are locally abundant and diverse, and show a variety of foraging strategies and habitat preferences. Both animal groups are sensitive to changes in habitat composition and structure, can be easily sampled and species level identification is usually not problematic. These reasons have led many researchers to use one or both of these arthropod groups as bioindicators in biodiversity and ecological studies (e.g. [41] and references therein).

We sampled the target groups by applying a standardized sampling protocol (the BALA protocol) which consists of setting 30 pitfall traps (plastic cups with 42mm wide and 78mm deep) spaced by 5m along a linear transect. The traps were filled in alternately with ~60ml of ethyleneglycol or Turquin solution [42] and a few drops of detergent to reduce the surface tension. A plastic plate was placed nearly 5cm above each trap to protect its contents from the rain and the traps remained active for a 15-day period. The BALA protocol proved to be both efficient and effective in sampling epigean terrestrial arthropods in Azorean island ecosystems, where it has been repeatedly used in inventory and monitoring programs [34, 35, 43]. The sampling took place during late spring and summer, the period when most spider and ground beetle species are active (S1 and S2 Tables). The standardized data on spider and ground beetle species richness and abundance used in this study were collected during several field surveys carried out in Terceira and Madeira. Forty sites were sampled in Terceira distributed across the five extant forest fragments (Biscoito da Ferraria, Caldeira Guilherme Moniz, Pico do Galhardo, Serra de Santa Bárbara and Terra Brava) while in Madeira sampling was carried out in 47 sites encompassing large and continuous forest patches, but also in smaller fragments (e.g. Funduras, Ribeira do Tristão and Ribeira da Cruz). We adopted a spatially extensive sampling design aiming to cover the diversity of habitats within Laurisilva and to obtain a representative sample of the spider and ground beetle diversity from each island. Furthermore, particularly in Madeira, we have also sampled more locally along altitudinal gradients. The complex geomorphology of Madeira (with steep slopes and deep valleys) posed some difficulties during sampling site selection since many areas are virtually inaccessible while in other locations we were not able to apply the BALA protocol (i.e. sampling along a 150m transect).

Pitfall samples were taken to the lab where the specimens were sorted and identified to species level. Taxonomic literature was used to identify the adult spiders [44–47] and ground beetles [48–50], and we also consulted the entomological collections of two public institutions (see below). The immature stages of both spiders and beetles are generally difficult to identify and were not considered in this study. All the specimens were deposited in the entomological collection of the Animal Biology Department (Faculty of Sciences, University of Lisbon, Portugal) and at the Dalberto Pombo entomological collection (University of Azores, Terceira, Portugal).

### Statistical analysis

Classical alpha diversity metrics following the Hill numbers were calculated for the four communities aiming to obtain a diversity profile organized in four orders (*q*) as follows: i) species richness (S) (*q* = 0), ii) the exponential Shannon-Wiener index (exp H´) (*q* = 1); iii) the inverse of Simpson´s concentration index (1/D) (*q* = 2) and iv) the Berger-Parker index (d)(*q* = 3). The Hill numbers are very informative since they combine knowledge on species richness, species rarity and species dominance and they are all expressed in the same units (i.e. effective number of species) making them comparable between each other [51–53]. In addition, to understand the level of completeness of our sampling, we calculated the Jackknife 1 non-parametric species richness estimator for the four communities since it is considered very robust to different sampling scales and conditions [54]. Then, sampling completeness was obtained using the ratio of observed species richness (S) over the Jackknife 1 estimation.

To further our understanding of the diversity patterns of spiders and ground beetles in Terceira and Madeira, we performed SAD and OFD analyses. The joint analysis of the variations in species abundances and species geographic range sizes provides a useful approach to identify patterns of commonness and rarity and is particularly helpful in comparative studies of (deconstructed) ecological assemblages. The performance of SAD and/or OFD analyses following the deconstruction of ecological assemblages into various subsets (e.g. native and introduced species) allows assessing the contribution of each subset to the overall pattern which, under a comparative framework, may provide valuable information for biodiversity management and conservation [21]. To evaluate variations in the shape of SADs of the Terceira and Madeira communities, we fitted two SAD models to the data: the logseries distribution, and the Poisson lognormal distribution (PLN; truncated form). These two models represent the most commonly observed empirical SAD shapes, and both have been found to provide good fits to empirical data [55]. The lognormal distribution is generally considered to accurately model the SAD of an undisturbed community, providing a helpful tool for measuring the impacts of disturbance on communities, while in many disturbed communities SADs have been shown to follow distributions close to the logseries [21]. However, a variety of patterns (including the opposite) have been recorded in empirical systems (see [21] and references therein). The PLN model was fitted using the ‘poilog’ R package [56] and, for each sample, the SAD models were compared using Akaike’s information criterion corrected for small sample size (AIC_c_) [57, 58]. The model with the smallest AIC_c_ value was considered as providing the best fit to the data. However, models with a delta AIC_c_ value lower than two (the difference between each model’s AIC_c_ and the lowest AIC_c_) were considered as having equal statistical support. In addition to comparing the fit of the PLN and the logseries for each dataset, we also applied the gambin model proposed by Ugland et al. [59], which has been shown to provide good fits to SADs [60]. To fit the gambin model we first binned the data into octaves (on a log 2 scale) and then we estimated for each dataset the standardized alpha parameter (α) of the gambin model which determines the shape of the distribution and the ‘dimensionality’ of the sampled community. Calculating α provided us with an additional metric with which to compare the shape of SADs between assemblages. These analyses were carried using the ‘gambin’ R package [60]. Initial examination of SAD plots revealed possible multimodality in the form of several SADs leading us to attempt to fit a two-mode PLN model [61]. However, as we were focused on subsets of taxa (e.g. ground beetles) there were issues related with the low number of collected specimens (particularly in Terceira) and the model did not converge in a number of instances. Thus, we do not present the results here.

The study of OFDs was carried out separately for epigean spiders and ground beetles from the islands of Madeira and Terceira. For each island, the number of sampling locations was subdivided into proportional classes (10 classes), each comprising 10% of the total number of sampling locations. Then, the cumulative number of species sharing the same frequency occupancy size class was plotted using frequency histograms. The analyses of modality in OFDs were done by applying the Tokeshi formulas following a two-step process [62]. First, under the null hypothesis of a uniform distribution, we tested the significance (*P* < 0.05) of the deviance from randomness of the overall shape of each frequency occupancy distribution (*P*_*c*_) aiming to identify the presence of modality in the data. Then, if the data presented a mode, we checked the significance (*P* < 0.05) of the peaks of the left-most (*P*_*l*_) and right-most (*P*_*r*_) classes. If the modes of both outermost classes (*P*_*l*_, *P*_*r*_) were significant the distribution was classified as bimodal, while in the case of only one mode being significant the OFD was considered to be unimodal. All modality tests were conducted using the original formulas [62] in a Microsoft Excel spreadsheet.

Potential rare species were identified following the quartile definition proposed by Gaston [18], which restricts the analysis of rarity to the species included in the first quartile of SADs and OFDs. This selection allowed us to produce a comprehensive list of potential rare species for both study islands. Then, taking into consideration the biology of each species and information from previous research on species-habitat associations in both archipelagos, a more detailed evaluation of rarity was carried out to distinguish between pseudo-rare (tourists and pseudo-rare microhabitat specialists) and truly rare species of the native laurel forests. We classified as tourist species those species that are rare in the Laurisilva, but are frequent and abundant in the surrounding habitats. Pseudo-rare microhabitat specialists are species characteristic of the Laurisilva, but occur in specific microhabitats (e.g. under the bark of trees, in tree holes, on the vegetation).

## Results

Sampling of epigean spiders and ground beetles in native forests yielded 1799 specimens from Terceira and 4971 from Madeira (Table 2). In Madeira, ground beetles were the dominant group (78.8% of specimens), whilst spiders were better represented in the samples from Terceira (48.1% of specimens). Species richness was considerably different between the two islands for both spiders (S = 21 in Terceira, S = 40 in Madeira) and ground beetles (S = 7 in Terceira, S = 34 in Madeira) and sampling completeness was similar for Madeira samples, but higher for spiders than ground beetles in the Azores (Tables 2 and 3). Introduced species of the two target groups were found in the native forests of both islands, but while they accounted for a large fraction of the fauna in Terceira (nearly half of the spider and ground beetle species), a much lower proportion of introduced species was reported in Madeira (15% of spiders and less than 9% of ground beetles). In fact, the Laurisilva of Madeira clearly contrasts with the native forests of Terceira by harbouring a larger proportion of indigenous species, particularly endemic ground beetles (Tables 2 and 3). Besides the differences in species richness and composition, the assemblages of spiders and ground beetles from the native forests of Terceira and Madeira are also structured differently. Epigean spiders showed similar abundance in native forests from Madeira (average abundance/site: 22.5 specimens) and Terceira (average abundance/site: 21.6 specimens), but considerable differences were recorded for ground beetles (Table 2). Terceira is extremely poor in regards to this insect group (average abundance/site: 23.3 specimens) when compared with Madeira (average abundance/site: 83.3 specimens). Hill numbers clearly showed a higher diversity of ground beetles and spiders in Madeira than in Terceira, and, surprisingly, a high dominance in Madeira spiders as reported by the Berger-Parker index (Table 2).

**Table 2:** Diversity metrics following the Hill numbers (*q*) and relative abundance of ground beetles and spiders from the native forests of Terceira and Madeira.

	Terceira	Madeira
**Ground beetles**		
Overall species richness (*q* = 0)	7	34
Estimated species richness (Jackknife 1)	10.9±2.3	44.8±3.5
Sample completeness	0.64	0.77
Exponential of Shannon-Wiener index (*q* = 1)	2.94	7.61
Simpson’s index (*q* = 2)	2.77	4.55
Berger-Parker index (*q* = 3)	0.47	0.32
Average species richness per site (and range)	0.7 (0–4)	5.1 (2–11)
Total number of individuals	933	3915
Average species abundance per site (and range)	23.3 (0–649)	83.3 (5–1051)
Proportion of introduced species	0.57	0.09
Proportion of endemic species	0.29	0.88
**Spiders**		
Overall species richness (*q* = 0)	21	40
Estimated species richness (Jackknife 1)	23.9±1.6	51.7±4.3
Sample completeness	0.88	0.77
Exponential of Shannon-Wiener index (*q* = 1)	8.17	11.13
Simpson’s index (*q* = 2)	2.78	4.54
Berger-Parker index (*q* = 3)	0.30	0.40
Average species richness per site (and range)	4.8 (2–9)	6.8 (0–15)
Total number of individuals	866	1056
Average species abundance per site (and range)	21.6 (4–148)	22.5 (0–79)
Proportion of introduced species	0.43	0.18
Proportion of endemic species	0.43	0.55

Data on species richness and abundance (overall and average per site) are presented jointly with the proportion of endemics and introduced species in each island for both study groups.

**Table 3:** Spider and ground beetle species richness, abundance and occupancy in Madeira and Terceira islands. List of the sampled spider and ground beetle species in Madeira and Terceira native forests with functional and taxonomic (family-level) information and indication of their overall abundance, occupancy and distributional status (END—endemic; NAT—native non-endemic; INT—introduced).

ISLAND	TAXONOMIC GROUP		SPECIES	DISTRIBUTION STATUS	OCCUPANCY	ABUNDANCE	FUNCTIONAL GROUP
	**Order**	**Family**					
Madeira	**Araneae**	**Linyphiidae**	***Centromerus variegatus* Denis**	**END**	**1**	**3**	sheet-web builder
Madeira	**Araneae**	**Linyphiidae**	***Ceratinopsis acripes* (Denis)**	**END**	**1**	**1**	sheet-web builder
Madeira	Araneae	Linyphiidae	*Ceratinopsis infuscata* (Denis)	END	14	22	sheet-web builder
Madeira	Araneae	Miturgidae	*Cheiracanthium albidulum* (Blackwall)	END	8	13	hunter
Madeira	Araneae	Clubionidae	*Clubiona decora* Blackwall	NAT	9	12	hunter
Madeira	Araneae	Theridiidae	*Cryptachaea blattea* (Urquhart)	INT	32	133	cobweb builder
Madeira	Araneae	Linyphiidae	*Diplostyla concolor* (Wider)	INT	3	5	sheet-web builder
Madeira	**Araneae**	**Theridiidae**	***Dipoenata longitarsis* (Denis)**	**END**	**1**	**1**	cobweb builder
Madeira	**Araneae**	**Dysderidae**	***Dysdera diversa* Blackwall**	**END**	**1**	**1**	hunter
Madeira	Araneae	Theridiidae	*Enoplognatha sattleri* Bösenberg	NAT	3	4	cobweb builder
Madeira	Araneae	Linyphiidae	*Entelecara schmitzii* Kulczynski	NAT	15	35	sheet-web builder
Madeira	Araneae	Theridiidae	*Episinus maderianus* Kulczynski	NAT	20	31	cobweb builder
Madeira	Araneae	Mimetidae	*Ero aphana* (Walckenaer)	INT	1	1	hunter
Madeira	Araneae	Linyphiidae	*Frontinellina dearmata* (Kulczynski)	END	2	3	sheet-web builder
Madeira	**Araneae**	**Linyphiidae**	***Frontiphantes fulgurenotatus* (Schenkel)**	**END**	**6**	**7**	sheet-web builder
Madeira	Araneae	Hahniidae	*Hahnia insulana* Schenkel	END	13	19	sheet-web builder
Madeira	Araneae	Dictynidae	*Lathys affinis* (Blackwall)	END	14	40	mesh-web builder
**Madeira**	**Araneae**	**Linyphiidae**	***Lepthyphantes impudicus* Kulczynski**	**END**	**2**	**3**	sheet-web builder
Madeira	Araneae	Linyphiidae	*Lepthyphantes lundbladi* Schenkel	END	2	13	sheet-web builder
Madeira	**Araneae**	**Linyphiidae**	***Lepthyphantes mauli* Wunderlich**	**END**	**1**	**1**	sheet-web builder
Madeira	Araneae	Salticidae	*Macaroeris diligens* (Blackwall)	NAT	11	22	hunter
Madeira	**Araneae**	**Salticidae**	***Macaroeris* n. sp.**	**END**	**2**	**2**	hunter
Madeira	Araneae	Gnaphosidae	*Macarophaeus cultior* (Kulczynski)	END	9	28	hunter
Madeira	**Araneae**	**Tetragnathidae**	***Meta stridulans* Wunderlich**	**END**	**3**	**4**	orb-web builder
Madeira	Araneae	Linyphiidae	*Microlinyphia johnsoni* (Blackwall)	NAT	1	1	sheet-web builder
Madeira	Araneae	Thomisidae	*Misumena spinifera* (Blackwall)	NAT	1	1	hunter
Madeira	Araneae	Theridiidae	*Paidiscura orotavensis* (Schmidt)	NAT	2	3	cobweb builder
Madeira	Araneae	Linyphiidae	*Palliduphantes schmitzi* (Kulczynski)	NAT	30	83	sheet-web builder
Madeira	Araneae	Philodromidae	*Philodromus insulanus* Kulczynski	END	1	2	hunter
Madeira	Araneae	Linyphiidae	*Poeciloneta variegata* (Blackwall)	INT	2	3	sheet-web builder
Madeira	Araneae	Theridiidae	*Rugathodes madeirensis* Wunderlich	END	21	33	cobweb builder
Madeira	Araneae	Theridiidae	*Steatoda nobilis* (Thorell)	NAT	1	1	cobweb builder
Madeira	Araneae	Linyphiidae	*Tenuiphantes tenebricoloides* (Schenkel)	END	3	31	sheet-web builder
Madeira	Araneae	Linyphiidae	*Tenuiphantes tenuis* (Blackwall)	INT	40	420	sheet-web builder
Madeira	Araneae	Tetragnathidae	*Tetragnatha intermedia* Kulczynski	INT	1	1	orb-web builder
Madeira	Araneae	Theridiidae	*Theridion melanurum* Hahn	INT	7	12	cobweb builder
Madeira	Araneae	Theridiidae	*Theridion* n. sp.	END	10	12	cobweb builder
Madeira	Araneae	Mysmenidae	*Trogloneta madeirensis* Wunderlich	END	15	36	3D orb-web builder
Madeira	Araneae	Linyphiidae	*Turinyphia maderiana* (Schenkel)	END	8	12	sheet-web builder
Madeira	Araneae	Araneidae	*Zygiella minima* Schmidt	NAT	1	1	orb-web builder
Madeira	Coleoptera	Carabidae	*Amara aenea* (De Geer)	INT	1	1	winged polyphagous
Madeira	Coleoptera	Carabidae	*Bradycellus assingi* Wrase & Jaeger	END	5	19	apterous generalist predator
Madeira	Coleoptera	Carabidae	*Bradycellus excultus* Wollaston	END	11	26	apterous generalist predator
Madeira	**Coleoptera**	**Carabidae**	***Bradycellus maderensis* Mateu**	**END**	**1**	**3**	apterous generalist predator
Madeira	Coleoptera	Carabidae	*Bradycellus wollastoni* Wrase & Jaeger	END	4	11	apterous generalist predator
Madeira	Coleoptera	Carabidae	*Calathus colasianus* Mateu	END	18	154	apterous generalist predator
Madeira	Coleoptera	Carabidae	*Calathus complanatus* Dejean	END	4	242	apterous generalist predator
Madeira	Coleoptera	Carabidae	*Calathus vividus* (Fabricius)	END	7	144	apterous generalist predator
Madeira	Coleoptera	Carabidae	*Cymindis maderae* Wollaston	END	1	2	apterous generalist predator
Madeira	Coleoptera	Carabidae	*Harpalus attenuatus* Stephens	NAT	1	1	winged polyphagous
Madeira	Coleoptera	Carabidae	*Loricera wollastoni* Javet	END	10	13	apterous specialist predator
Madeira	Coleoptera	Carabidae	*Nesarpalus gregarius* (Fauvel)	END	1	1	apterous generalist predator
Madeira	Coleoptera	Carabidae	*Olisthopus ericae* Wollaston	END	4	7	apterous generalist predator
Madeira	Coleoptera	Carabidae	*Olisthopus maderensis* Wollaston	END	1	1	apterous generalist predator
Madeira	Coleoptera	Carabidae	*Orthomus annae* (Donabauer)	END	9	99	apterous generalist predator
Madeira	Coleoptera	Carabidae	*Orthomus berrai* (Battoni)	END	6	42	apterous generalist predator
Madeira	Coleoptera	Carabidae	*Orthomus curtus* (Wollaston)	END	31	1245	apterous generalist predator
Madeira	Coleoptera	Carabidae	*Orthomus dilaticollis* (Wollaston)	END	12	1244	apterous generalist predator
Madeira	Coleoptera	Carabidae	*Orthomus gracilipes* (Wollaston)	END	23	137	apterous generalist predator
Madeira	Coleoptera	Carabidae	*Orthomus lundbladi* (Jeannel)	END	1	12	apterous generalist predator
Madeira	Coleoptera	Carabidae	*Paradromius insularis* (Wollaston)	END	1	1	apterous generalist predator
Madeira	Coleoptera	Carabidae	*Scarites abbreviatus* Dejean	END	37	205	apterous generalist predator
Madeira	Coleoptera	Carabidae	*Trechus custos* Wollaston	END	2	5	apterous generalist predator
Madeira	Coleoptera	Carabidae	*Trechus decolor* Jeannel	END	6	63	apterous generalist predator
Madeira	Coleoptera	Carabidae	*Trechus dilutus* Wollaston	END	3	13	apterous generalist predator
Madeira	Coleoptera	Carabidae	*Trechus flavomarginatus* Wollaston	END	2	17	apterous generalist predator
Madeira	Coleoptera	Carabidae	*Trechus fulvus* Dejean	INT	1	1	apterous generalist predator
Madeira	**Coleoptera**	**Carabidae**	***Trechus maderensis* Csiki**	**END**	**1**	**2**	apterous generalist predator
Madeira	**Coleoptera**	**Carabidae**	***Trechus minyops* Wollaston**	**END**	**5**	**6**	apterous generalist predator
Madeira	**Coleoptera**	**Carabidae**	***Trechus nigrocruciatus* Wollaston**	**END**	**1**	**1**	apterous generalist predator
Madeira	Coleoptera	Carabidae	*Trechus nugax* Lompe	END	5	23	apterous generalist predator
Madeira	Coleoptera	Carabidae	*Trechus obtusus* Erichson	INT	5	18	winged generalist predator
Madeira	Coleoptera	Carabidae	*Trechus umbricola* Wollaston	END	18	152	apterous generalist predator
Madeira	**Coleoptera**	**Carabidae**	***Zargus schaumii* Wollaston**	**END**	**3**	**4**	apterous generalist predator
Terceira	Araneae	Linyphiidae	*Acorigone acoreensis* (Wunderlich)	END	4	10	sheet-web builder
Terceira	Araneae	Linyphiidae	*Agyneta decora* (O.P. Cambridge)	INT	7	13	sheet-web builder
Terceira	Araneae	Linyphiidae	*Canariphantes acoreensis* (Wunderlich)	END	17	36	sheet-web builder
Terceira	Araneae	Dysderidae	*Dysdera crocata* C.L. Koch	INT	10	21	Hunter
Terceira	Araneae	Linyphiidae	*Erigone atra* Blackwall	INT	3	3	sheet-web builder
Terceira	Araneae	Linyphiidae	*Erigone autumnalis* Emerton	INT	1	2	sheet-web builder
Terceira	Araneae	Mimetidae	*Ero furcata* (Villers)	INT	7	11	Hunter
Terceira	Araneae	Linyphiidae	*Mermessus bryantae* (Ivie & Barrows)	INT	2	2	sheet-web builder
Terceira	Araneae	Linyphiidae	*Minicia floresensis* Wunderlich	END	2	5	sheet-web builder
Terceira	Araneae	Linyphiidae	*Oedothorax fuscus* (Blackwall)	INT	2	2	sheet-web builder
Terceira	Araneae	Linyphiidae	*Palliduphantes schmitzi* (Kulczynski)	NAT	12	40	sheet-web builder
Terceira	Araneae	Lycosidae	*Pardosa acorensis* Simon	END	22	256	Hunter
Terceira	Araneae	Pisauridae	*Pisaura acoreensis* Wunderlich	END	5	6	Hunter
Terceira	Araneae	Linyphiidae	*Porrhomma borgesi* Wunderlich	END	10	15	sheet-web builder
Terceira	Araneae	Theridiidae	*Rugathodes acoreensis* Wunderlich	END	21	107	tangle- or cobweb builder
Terceira	Araneae	Tetragnathidae	*Sancus acoreensis* (Wunderlich)	END	1	1	orb-web builder
Terceira	Araneae	Linyphiidae	*Tenuiphantes miguelensis* Wunderlich	NAT	38	202	sheet-web builder
Terceira	Araneae	Linyphiidae	*Tenuiphantes tenuis* (Blackwall)	INT	17	102	sheet-web builder
Terceira	Araneae	Linyphiidae	*Walckenaeria grandis* (Wunderlich)	END	5	11	sheet-web builder
Terceira	Araneae	Thomisidae	*Xysticus cor* Canestrini	NAT	5	18	Hunter
Terceira	Araneae	Thomisidae	*Xysticus nubilus* Simon	INT	1	3	Hunter
Terceira	Coleoptera	Carabidae	*Amara aenea* (De Geer)	INT	1	1	winged polyphagous
Terceira	Coleoptera	Carabidae	*Anisodactylus binotatus* (Fabricius)	INT	1	1	winged polyphagous
Terceira	Coleoptera	Carabidae	*Cedrorum azoricus azoricus* Borges & Serrano	END	9	186	apterous generalist predator
Terceira	Coleoptera	Carabidae	*Ocys harpaloides* (Audinet-Serville)	NAT	1	1	winged generalist predator
Terceira	Coleoptera	Carabidae	*Paranchus albipes* (Fabricius)	INT	8	436	winged generalist predator
Terceira	Coleoptera	Carabidae	*Pterostichus vernalis* (Panzer)	INT	1	2	winged generalist predator
Terceira	Coleoptera	Carabidae	*Trechus terrabravensis* Borges, Serrano & Amorim	END	8	306	apterous generalist predator

The distributional status follows [30] for Madeiran species and [31] for the Azorean ones. Rare species are highlighted in bold.

**Fig 1:**
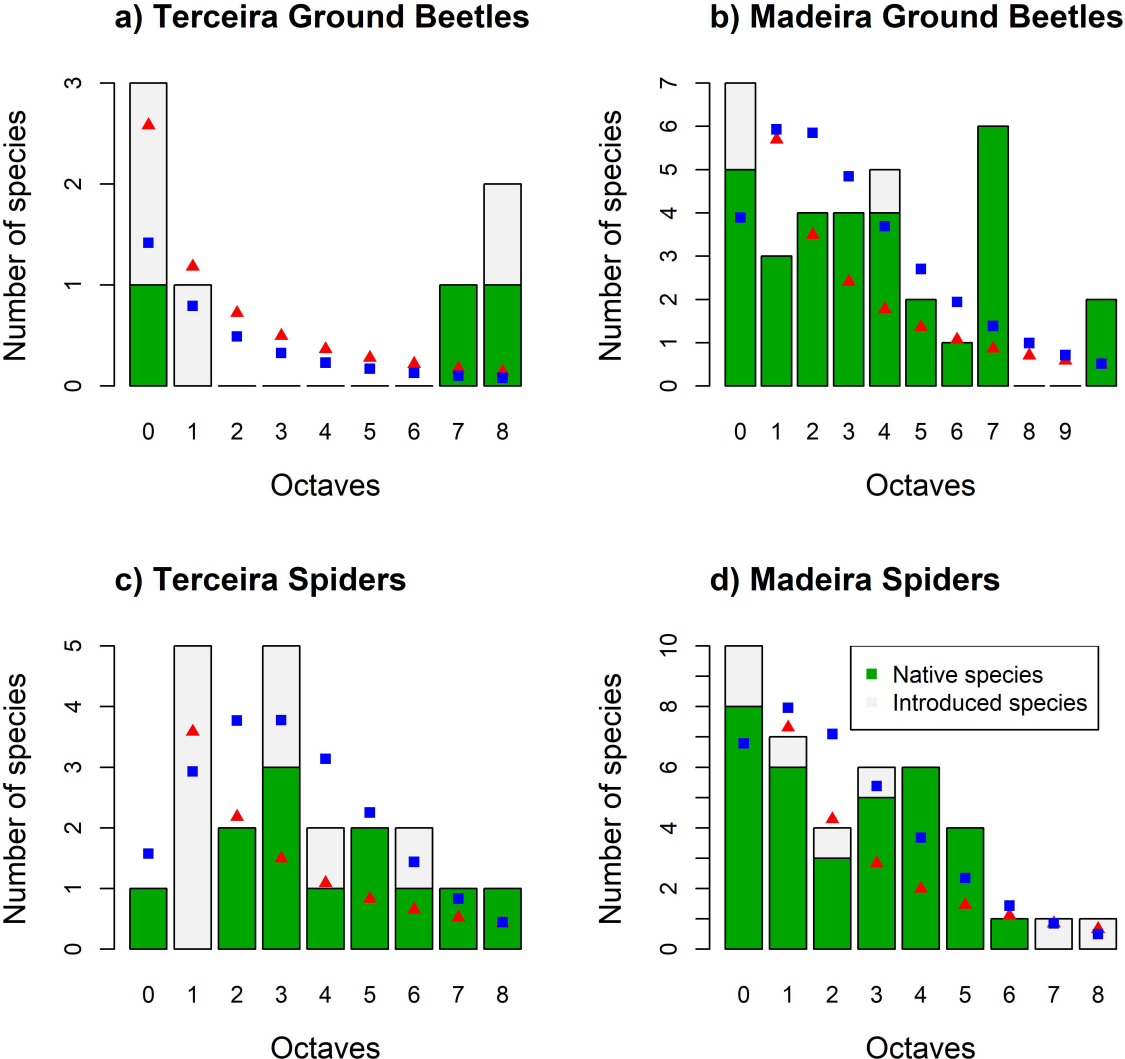
Species abundance distributions (SADs) for spiders and ground beetles from the native forests of Terceira and Madeira islands. The best fit models (logseries in red triangles and PLN in blue squares) are plotted together with the empirical data. The number of native species is presented in green while the number of introduced species is shown in white. (A) SAD of Terceira ground beetles. (B) SAD of Madeira ground beetles. (C) SAD of Terceira spiders. (D) SAD of Madeira spiders.

The comparative analyses of SADs highlight considerable differences between the assemblages of ground beetles from the two islands. The remnants of native forest in Terceira supported a depauperate ground beetle fauna, where on average less than one species was found per site (0.7 species/site). One introduced species (*Paranchus albipes*) was co-dominant with two less abundant endemic species (*Cedrorum azoricus* and *Trechus terrabravensis*) and the remaining species, mainly introduced species, were found in scarce numbers (Fig 1A and Table 3). The ground beetle assemblages from Madeira Laurisilva were more species-rich, being dominated by endemic species (particularly *Orthomus curtus* and *O*. *dilaticollis*), and introduced species were seldom found. The lack of ground beetle representatives in the intermediate abundance classes of Terceira contrasts markedly with the SAD pattern found in Madeira (Fig 1B). The SADs of spiders from Terceira and Madeira showed a similar pattern, where all abundance classes had representatives, less abundant species were the dominant group and only a small number of abundant species was recorded (Fig 1C). Curiously, the spider assemblages from Terceira included a higher proportion of introduced species (43%), but in Madeira the lower percentage of introduced species (18%) accounted for over 54% of overall species abundance, with the introduced species *Tenuiphantes tenuis* and *Cryptachaea blattea* being the most abundant spiders. Nevertheless, most of the introduced spiders in Madeira have low abundance while in the Azores they are included in a range of abundance classes.

For both spiders and ground beetles, in both study islands, the best fitting SAD model was the logseries (Table 4); however, closer examination of SAD plots (Fig 1) revealed differences between the observed SADs that were not apparent when just comparing the fit of the logseries and the PLN models. For example, for ground beetles in Terceira, the SAD exhibited a bimodal shape contrasting markedly with the SAD pattern found for Madeiran beetles, whilst for spiders the Terceira SAD had a much lower proportion of singleton species when compared with the findings for Madeira. In regards to the gambin model, the standardised α values for ground beetles were 0.64 for Terceira and 2.18 for Madeira, whilst the α values for spiders were 3.98 for Terceira and 1.83 for Madeira (see also Fig 1).

**Table 4:** Model selection results for the species abundance distributions.

Taxonomic group	Island	PLN	Logseries
Ground beetles	Terceira	72.56	**67.16**
	Madeira	328.48	**324.67**
Spiders	Terceira	188.62	**187.69**
	Madeira	302.42	**299.58**

Goodness of fit and model selection results for the species abundance distributions of spiders and ground beetles from the native forests of Terceira and Madeira islands. The Akaike’s information criterion corrected for small sample size (AIC_c_) is given for the two distributions—Logseries and Poisson lognormal (PLN)—and the model representing the best fit is highlighted in bold.

**Fig 2:**
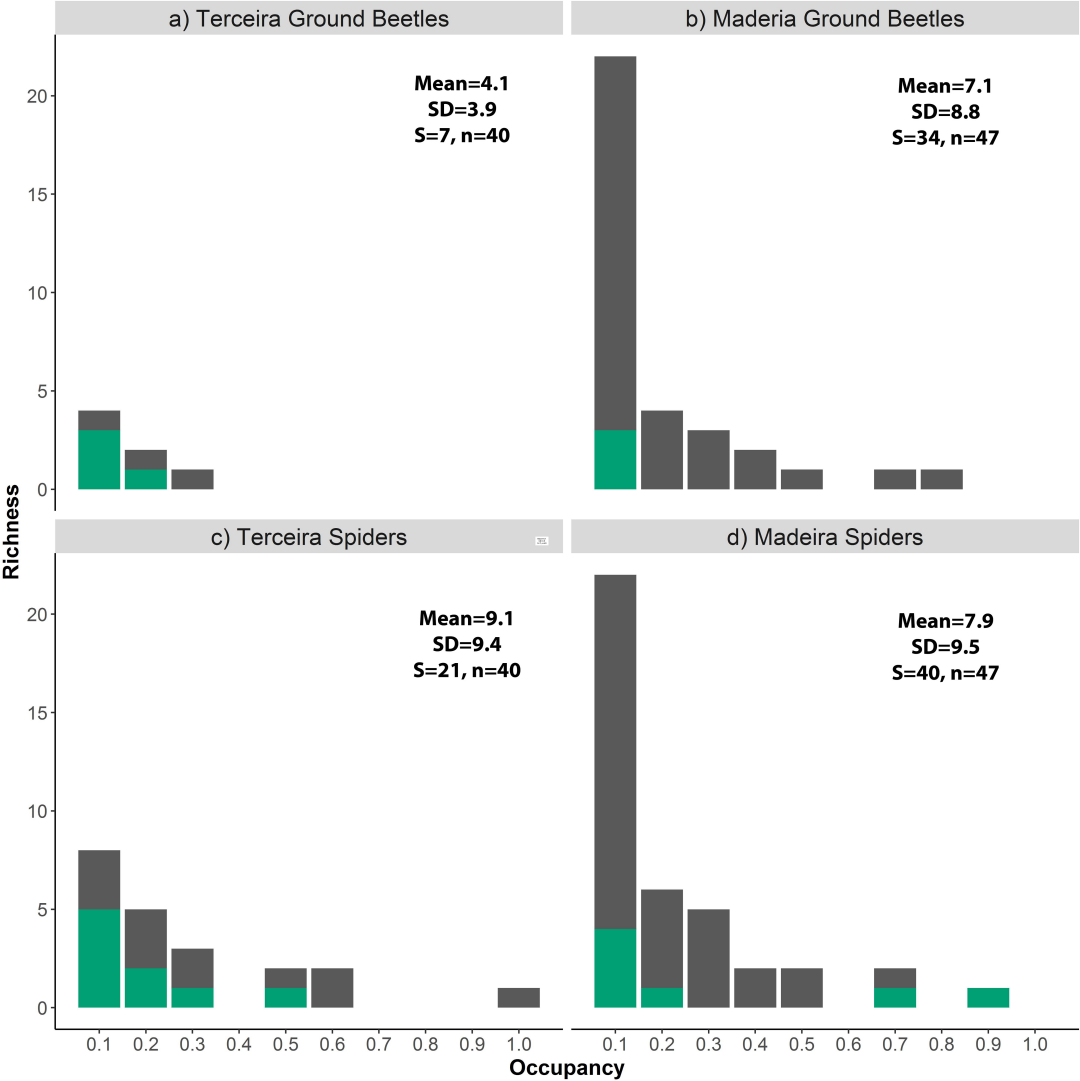
Occupancy-frequency distributions (OFDs) for spiders and ground beetles from the native forests of Terceira and Madeira islands. Overall species richness per occupancy class is shown in grey and the contribution of introduced species is highlighted in green. The mean and standard deviation of regional occupancy is shown together with the overall species richness (S) and the number of sampling units (n). (A) OFD of Terceira ground beetles. (B) OFD of Madeira ground beetles. (C) OFD of Terceira spiders. (D) OFD of Madeira spiders.

The OFDs of epigean spiders and ground beetle assemblages from Madeira Laurisilva were strongly right-skewed (Fig 2, Table 5), indicating that most species are confined to a few locations. Only a few species were found in a high number of sampling locations (i.e. >60% of the sites): the abovementioned introduced spiders–*T*. *tenuis* and *C*. *blattea*–, the native spider *Palliduphantes schmitzi* and the endemic ground beetles *Orthomus curtus* and *Scarites abbreviatus*. In Terceira, most species of the two target groups were characterised by low occupancy, but the OFDs were less strongly right-skewed relative to those based on the Madeira data (Fig 2, Table 5). The unusual OFD for ground beetles, where only a few species can be found in a low number of sites, clearly highlights the species poor fauna of Terceira. Interestingly, the OFDs of introduced species in Terceira match the pattern observed for native species while in Madeira they are locally restricted (with the two exceptions previously reported in spiders).

**Table 5:** Modality of occupancy frequency distributions.

Taxonomic group	Island	*P*_*c*_	*P*_*l*_	*P*_*r*_	Distribution
Ground beetles	Terceira	<0.003	< 0.001	0.52	Unimodal
Ground beetles	Madeira	< 0.001	< 0.001	0.97	Unimodal
Spiders	Terceira	< 0.001	< 0.001	0.64	Unimodal
Spiders	Madeira	< 0.001	< 0.001	0.98	Unimodal

Tokeshi test results for the modality of occupancy frequency distributions on spider and ground-beetle data from Madeira and Terceira (*P*_*c*_, *P*_*l*_, and *P*_*r*_ represent the results of the overall, left-, and right-most class modality tests, respectively).

**Fig 3:**
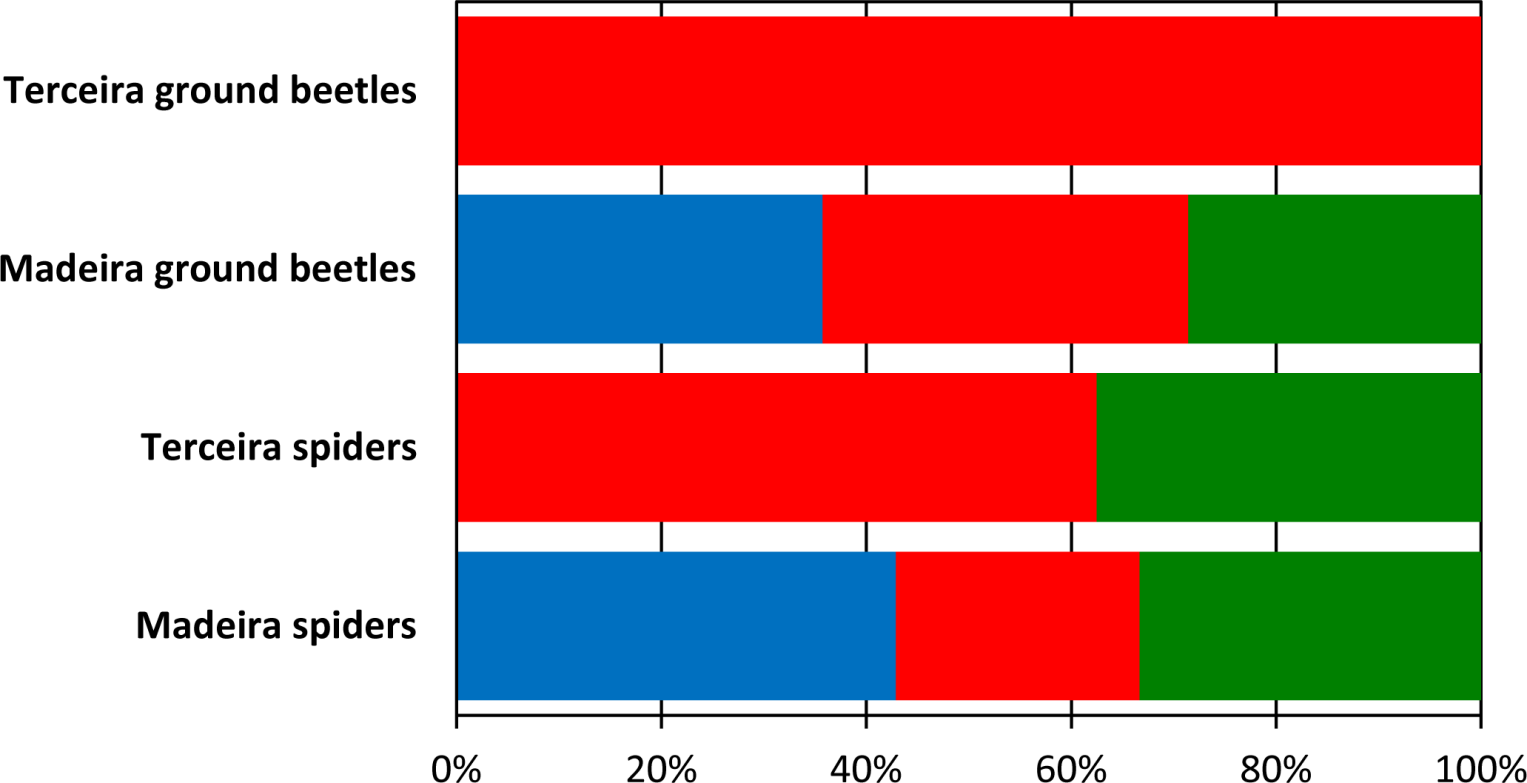
Evaluation of potential rare spider and ground beetle species from the native forests of Terceira and Madeira. Potential rare species (i.e. low-abundance and range-restricted) were classified as tourists (red), microhabitat pseudo-rare species (green) and truly rare species (blue). The number of evaluated species (n) from each island and study group is also shown.

Finally, the analysis of species rarity in groups of low-abundance range-restricted (i.e. potential rare) spiders and ground beetles from the native forests of Madeira and Terceira enabled the discrimination between pseudo-rare and true rare species. In Terceira all potential rare species were found to be pseudo-rare and include species that are common in neighbouring habitats (tourists), but also forest microhabitat specialists (e.g. arboreal specialist species) that are not adequately sampled by the adopted sampling methodology (Fig 3). In Madeira, 40% of the evaluated species were considered truly rare, with a slightly higher number of rare spider species (S = 9) than ground beetles (S = 5). The rare ground beetles include the endemics *Bradycellus madeirensis*, *Zargus schaumii* and three *Trechus* species (Table 3). Other endemic ground beetles, like *Olisthopus maderensis* and *Paradromius insularis*, were not considered rare because they are microhabitat specialists living under the bark of the trees and seldom being found at the soil surface [63]. All the spiders classified as truly rare are also Madeira endemics and include *Centromerus variegatus*, *Ceratinopsis acripes*, *Dipoenata longitarsis*, *Dysdera diversa*, *Frontiphantes fulgurenotatus*, *Meta stridulans*, two *Lepthyphantes* species and one undescribed *Macaroeris* species (Table 3). Nearly a third of the evaluated spider species were classified as pseudo-rare microhabitat specialists since they are associated with the vegetation/canopy, where they are common, but are rarely found at ground level. This group of species includes, for example, the endemic linyphiid *Frontinellina dearmata* and the native thomisid *Misumena spinifera*. Finally, an observation worth highlighting is the absence of truly rare species and the much higher proportion of tourist species in Terceira native forests in comparison with the findings from the native laurel forests of Madeira (Fig 3).

## Discussion

The comparative analysis of spider and ground beetle assemblages from Terceira and Madeira native forests showed striking differences in species richness and composition between the two islands, which to some extent relate to the differences in colonization history and species diversification, but also reflect the intensity of the more recent human-mediated impacts on the native forests. The archipelagos of the Azores and Madeira differ considerably in some geographical, geological and ecological attributes that are important determinants of the biodiversity of the islands [26, 28, 29]. In fact, the relatively recent origin of the Azorean islands, their large distance to potential colonization sources and the low habitat diversity have been pointed out as the main drivers of the biodiversity observed in these islands [64]. Madeira is characterised by a more complex geomorphology that has favoured allopatric diversification events in some lineages [30], and due to its proximity to the paleoislands of the Canarian and Madeiran volcanic provinces, island colonization by a stepping-stone process was certainly eased [27]. Consequently, we expected to find a higher number of spider and ground beetle species associated with native laurel forests in Madeira than in Terceira. The recent history of the two archipelagos, following human colonization in the 15^th^ century, is marked by severe destruction and fragmentation of Azorean native forests while in Madeira some large pristine forest patches have remained [26–28]. Our findings on the composition and structure of spider and ground beetle assemblages reveal major differences in the severity of the impact of human-mediated changes between the two study islands, which can be inferred from the much larger proportion of introduced species found in Terceira native forests. Further, notably for ground beetles, SADs and OFDs are atypical and indicative of the severe disturbance effects on species richness, abundance and distribution echoing dramatic species extinctions [15]. The lack of intermediate abundance classes in Terceira SADs (Fig 1A), combined with the high proportion of introduced species in most abundance classes (Fig 1C), is indicative of disturbed communities, and clearly contrasts with the results from Madeira Laurisilva where native species are dominant and introduced species are uncommon (Fig 1B and 1D). The high abundance and occupancy of two introduced spiders in Madeira Laurisilva are an exception to this pattern and the reasons for their ecological success merits further investigation. We hypothesize that these species may have benefited from the lower biophysical resistance in Madeira Laurisilva (than in Terceira) since the structure of this forest is relatively similar to the habitat structure of their areas of origin. Furthermore, human colonization of inland Madeira is more ancient than in Terceira allowing more opportunities for these species to spread and the recent effects of global warming, more evident in Madeira than Terceira, may have also benefited their invasion. It is also important to stress that the invasion of Terceira Laurisilva by *T*. *tenuis* is probably being prevented by its endemic congener *T*. *miguelensis* that remains abundant and widespread in these forests while in Madeira Laurisilva no dominant native species seems to share similar traits with *T*. *tenuis*. Thus, a trait-based approach will be critical to clarify the reasons for the ecological dominance of these two introduced species in Madeira Laurisilva.

The patterns of SADs and OFDs of introduced species in Terceira match the ones found for the native assemblages suggesting that introduced species are well integrated within natural forest communities, most probably as a consequence of the long history of disturbance events in Azorean native forests [25]. It must also be emphasized that the young age and the isolation of the Azorean islands resulted in unsaturated local communities which are thought to offer greater opportunities for introduced species to spread and establish, especially when the natural habitat is disturbed as in Terceira Island [65]. Therefore, the observed SADs of Terceira may be the consequence of both historical and contemporary factors that render native forests more vulnerable to the effects of species introductions on community structure and composition.

For both spiders and ground beetles, in both study islands, the best fitting SAD model was the logseries (Table 4). However, these model comparisons do not clarify some of the differences in the observed SADs that were more apparent when comparing SAD plots and gambin’s α values. For instance, the joint analysis of SADs (Fig 1) and gambin’s α values indicates that, in Madeira, ground beetles have a much higher proportion of species in intermediate abundance classes, whilst a larger proportion of spiders are low abundant species. Deconstructing the assemblages allowed for further inferences to be made regarding the observed SADs (Fig 1). For example, despite all SADs being best fit by the logseries model, and thus being characterised by a relatively high number of singleton and doubleton species, further analysis revealed that in Madeira most of these low abundant species are endemics, whilst in the Azores the low and intermediate abundance species are often introduced species. Thus, although classical SAD hypotheses would suggest that Terceiran and Madeiran assemblages should be best fitted by different SAD models, due to differences in disturbance regimes between the two islands, our results show that such hypotheses are not always applicable in real world island systems [21]. Many island endemic invertebrate species are naturally present in low numbers, and thus the SADs of these assemblages in low disturbed systems (e.g. Madeira) are often characterised by more rare species than predicted by the logseries and ecological theory. On the other hand, in highly disturbed systems (e.g. Terceira), the presence of tourist species may inflate the number of species in low and intermediate abundance classes and mask the loss of native forest species [66]. The species-poor bimodal SAD observed for ground beetles in Terceira shows the consequences of previous extinctions of native forest species [15, 25] which have resulted in an unbalanced community with a few dominant species and several rare species. Indeed, previous research work on Azorean biodiversity has documented the role of human activities on the disturbance of native forests, the loss of native species and the dominance of some introduced species in natural forests [15, 67, 68].

The observed discrepancy in the diversity patterns among ground beetles and spiders in the native forests of Terceira seems to illustrate the differences in their vulnerability to disturbance. Nevertheless, the response of ground beetles and spiders to forest habitat disturbance is species-specific and depends mostly on the degree of habitat specialization, but also on dispersal ability and hunting strategy. Many island native ground beetles are flightless species with poor dispersal capacity, being less able to move away from the disturbed areas or even escape from alien predators [69, 70]. Spiders, in general, have higher dispersal capability through ballooning and their cryptic habitats may render them less susceptible to alien predators. Furthermore, several studies highlight that specialist ground beetles are extremely vulnerable to changes in microhabitat, particularly soil humidity, soil micro-topography, degree of canopy cover, leaf litter amount and the availability of decaying wood ([71, 72] and references therein). Thus, the high number of reported local extinctions of forest specialist ground beetles during the last decades as a consequence of human disturbance is not surprising (e.g. [72, 73]).

A recent study on the Azorean endemic beetle records concluded that at least seven species went extinct since the first reliable species records from the Azores, nearly 150 years ago [15]. This study allowed a crude estimation of an extinction rate of 4.96 species/century in the Azores just for endemic beetles, which is clearly indicative of the generalised and serious human impact on native biodiversity since the second half of the 19^th^ century. However, a much higher number of species was certainly lost during the previous four centuries following human colonization of the archipelago when more severe and extensive native habitat destruction took place (see [27, 74] and references therein). Scientific evidence of considerable changes in the biodiversity of the Azores (and to a less extent in Madeira) associated to human colonization of the archipelago comes directly from several historical reports, pollen analysis and studies on the fossil bird and land snail faunas, and indirectly from suspected extinctions on specific arthropod genera [11, 67, 74–78].

The greater vulnerability to extinction of narrow-range specialist species at higher trophic levels has been stressed in various empirical studies when assessing the effects of human impact on ecosystems (e.g. [79–81]). For instance, several studies showed that forest specialist ground beetles are virtually absent from disturbed or small forest patches where viable populations cannot persist, occurring exclusively in large undisturbed forest areas ([82] and references therein). Furthermore, many forest specialist species lack functional wings (a common feature in Azorean and Madeiran native forest endemics; see Table 3) making the possibility of re-colonization of isolated forest patches after local extinction events very unlikely and rendering these species extremely vulnerable to alien predators and competitors [69, 70]. Actually, most of the recently reported beetle extinctions in Azores are narrow-range flightless forest specialists and some of the currently threatened terrestrial arthropods in Macaronesia share these same characteristics [15].

The analysis of species rarity in groups of low-abundance range-restricted spiders and ground beetles from the native laurel forests of Madeira and Terceira highlighted substantial differences between the two study islands. The absence of rare native species coupled with the presence of a high number of tourists (mostly introduced species) in Terceira’s laurel forests suggests the past extirpation of populations of the most vulnerable native species. It is widely recognized that low abundance and narrow distribution are drivers that predispose species to extinction and both factors have already been associated with previous extinctions in a variety of animal and plant taxa [7, 18, 79, 83]. However, of note was the finding that in Terceira’s forests the loss of native species seems to have been balanced by the colonization of introduced species from the neighbouring disturbed habitats. These introduced species are abundant in the surrounding matrix of deforested man-made habitats (dominated by pastures), have good dispersal capacity (e.g. winged species) and may benefit from constant dispersal from disturbed areas that is sufficient to maintain viable populations in the native forest [22]. Several theoretical and empirical studies have shown that introduced species, even if they are inferior competitors, may succeed in invaded habitats simply by benefiting from the susceptibility of native species to habitat fragmentation and their lower ability to reinforce declining populations [79, 84, 85]. The homogenization of Azorean laurel forests due to the loss of rare native species and the establishment of exotics is a serious conservation problem that needs further research and the adoption of effective measures to halt biodiversity loss [66, 86]. The high levels of extinction debt found in endemic forest-dependent species [25], particularly for beetles and spiders, further highlights how acute this situation is, and the urgent need to implement large-scale conservation efforts in the archipelago.

In contrast with the findings in Terceira, the potential rare species in Madeira Laurisilva are largely true rare species (14 endemic species), but pseudo-rare microhabitat specialists (only native species) are also well represented. The low presence of tourist species in Madeira Laurisilva, combined with the small number of introduced species, illustrates the favourable conservation status of these forests. Nevertheless, it must be emphasized that Madeira Laurisilva has also suffered a considerable destruction in the past which, in combination with invasive species introductions, has led to the loss of some endemic species [11, 76, 77].

In conclusion, Madeira and the Azores have both been affected by human-mediated activities during the last few centuries which have altered the biodiversity in both islands. However, our comparative study on the spider and ground beetle assemblages of native forests in Madeira and Terceira has highlighted considerable differences in community structure and composition that mirror the differences in severity of human-induced changes between the two islands. The high proportion of introduced species, the virtual absence of rare native species and the finding that SADs and OFDs of introduced species match the pattern of native species in Terceira reinforce the role of man as an important driver of species diversity in oceanic islands, and provide additional evidence of the extensive and severe human-induced loss of the indigenous diversity of Terceira native forests that cannot be fully understood based on the current knowledge on species extinctions. The performance of comparative studies on the community structure and composition of island arthropods addressing the relative contribution of true rare endemics and introduced species to island biodiversity patterns can be very useful to evaluate the extent of species loss because “to neglect such extinctions is to ignore the majority of species that are or were in need of conservation” [7].

## Supporting information

S1 Table Sampling sites from Terceira island with indication of the geographic coordinates (in decimal degrees) and altitude (in meters) jointly with information on the sampling period. (DOC)

S2 Table Sampling sites from Madeira island with indication of the geographic coordinates (in decimal degrees) and altitude (in meters) jointly with information on the sampling period. (DOC)
