# Supplementary material for: A comparative analysis of terrestrial arthropod assemblages from a relict forest unveils historical extinctions and colonization differences between two oceanic islands
Source: PLoS One. 2018 Apr 25;13(4):e0195492. doi: 10.1371/journal.pone.0195492 (PMC5918893; doi:10.1371/journal.pone.0195492)
Supplement: S1 Table — (DOC) [file pone.0195492.s001.doc]

S1 Table – Sampling sites from Terceira island with indication of the geographic coordinates (in decimal degrees) and altitude (in meters) jointly with information on the sampling period.

| **Site** | **Latitude** | **Longitude** | **Altitude** | **Sampling period** |
| --- | --- | --- | --- | --- |
| Biscoito da Ferraria t1 | 38.7613 | -27.2204 | 693 | June 1999 |
| Biscoito da Ferraria t2 | 38.7583 | -27.2374 | 574 | June 1999 |
| Biscoito da Ferraria t3 | 38.7562 | -27.2094 | 775 | August 2003 |
| Biscoito da Ferraria t38 | 38.7644 | -27.2213 | 681 | June 2003 |
| Biscoito da Ferraria tp39 | 38.7570 | -27.2302 | 587 | June 2003 |
| Biscoito da Ferraria tp41 | 38.7535 | -27.2088 | 686 | June 2003 |
| Biscoito da Ferraria tp42 | 38.7599 | -27.2294 | 570 | July 2003 |
| Biscoito da Ferraria ty4 | 38.7550 | -27.2363 | 580 | July 2003 |
| Caldeira Guilherme Moniz t23 | 38.7088 | -27.2037 | 459 | July 2002 |
| Caldeira Guilherme Moniz t53 | 38.7051 | -27.2130 | 464 | September 2003 |
| Caldeira Guilherme Moniz t54 | 38.7115 | -27.2083 | 463 | September 2003 |
| Caldeira Guilherme Moniz ty35 | 38.7115 | -27.2152 | 467 | September 2003 |
| Pico Galhardo t40 | 38.7332 | -27.2286 | 648 | August 2003 |
| Pico Galhardo t44 | 38.7358 | -27.2276 | 601 | July 2003 |
| Pico Galhardo tg22 | 38.7364 | -27.2340 | 589 | July 2007 |
| Pico Galhardo tg33 | 38.7340 | -27.2272 | 651 | July 2007 |
| Serra de Santa Barbara t10 | 38.7347 | -27.3122 | 930 | August 1999 |
| Serra de Santa Barbara t11 | 38.7589 | -27.3091 | 623 | July 2001 |
| Serra de Santa Barbara t164 | 38.7358 | -27.3083 | 890 | July 2008 |
| Serra de Santa Barbara t43 | 38.7642 | -27.3145 | 536 | July 2003 |
| Serra de Santa Barbara t46 | 38.7334 | -27.2836 | 613 | July 2003 |
| Serra de Santa Barbara t47 | 38.7365 | -27.3119 | 935 | July 2003 |
| Serra de Santa Barbara t57 | 38.7571 | -27.3086 | 705 | July 2003 |
| Serra de Santa Barbara t6 | 38.7506 | -27.3328 | 786 | June 1999 |
| Serra de Santa Barbara t7 | 38.7391 | -27.2911 | 693 | July 1999 |
| Serra de Santa Barbara t9 | 38.7353 | -27.3081 | 879 | August 1999 |
| Serra de Santa Barbara te45 | 38.7386 | -27.2948 | 735 | July 2003 |
| Serra de Santa Barbara te48 | 38.7512 | -27.3318 | 748 | July 2003 |
| Serra de Santa Barbara te49 | 38.7475 | -27.3189 | 930 | July 2003 |
| Serra de Santa Barbara te50 | 38.7550 | -27.3173 | 702 | August 2003 |
| Serra de Santa Barbara ty11 | 38.7346 | -27.3120 | 912 | July 2003 |
| Serra de Santa Barbara ty5 | 38.7259 | -27.3262 | 822 | June 2003 |
| Terra Brava t16 | 38.7363 | -27.2062 | 673 | June 1999 |
| Terra Brava t173 | 38.7350 | -27.2006 | 676 | July 2008 |
| Terra Brava t18 | 38.7323 | -27.1980 | 668 | June 2002 |
| Terra Brava t51 | 38.7378 | -27.2068 | 650 | September 2003 |
| Terra Brava t55 | 38.7317 | -27.2075 | 710 | September 2003 |
| Terra Brava t56 | 38.7360 | -27.1988 | 641 | September 2003 |
| Terra Brava tg34 | 38.7381 | -27.2017 | 642 | July 2007 |
| Terra Brava ty15 | 38.7352 | -27.2021 | 639 | June 2003 |
