# Supplementary material for: A comparative analysis of terrestrial arthropod assemblages from a relict forest unveils historical extinctions and colonization differences between two oceanic islands
Source: PLoS One. 2018 Apr 25;13(4):e0195492. doi: 10.1371/journal.pone.0195492 (PMC5918893; doi:10.1371/journal.pone.0195492)
Supplement: S2 Table — (DOC) [file pone.0195492.s002.doc]

S2 Table – Sampling sites from Madeira island with indication of the geographic coordinates (in decimal degrees) and altitude (in meters) jointly with information on the sampling period.

| **Site** | **Latitude** | **Longitude** | **Altitude** | **Sampling period** |
| --- | --- | --- | --- | --- |
| Achada do Teixeira – norte | 32.7762 | -16.9022 | 1103 | May 2006 |
| Achada do Teixeira – sul | 32.7733 | -16.9081 | 1211 | May 2006 |
| Caramujo | 32.7746 | -17.0559 | 1001 | June 2007 |
| Caramujo – Casa do Levadeiro | 32.7722 | -17.0529 | 981 | June 2007 |
| Casa do Elias | 32.8268 | -17.1883 | 814 | June 2007 |
| Chão da Ribeira | 32.7933 | -17.1122 | 519 | May 2006 |
| Chão da Ribeira – Viveiro das Trutas | 32.7957 | -17.1117 | 491 | May 2006 |
| Chão dos Louros | 32.7636 | -17.0190 | 748 | May 2006 |
| Chão dos Louros – Parque | 32.7602 | -17.0141 | 825 | May 2011 |
| Curral Falso | 32.8062 | -17.1409 | 1134 | May 2006 |
| Encumeada | 32.7558 | -17.0143 | 999 | May 2006 |
| Fajã da Nogueira – Casa do Levadeiro | 32.7406 | -16.9136 | 989 | May 2006 |
| Fajã da Nogueira – Tanque | 32.7425 | -16.9168 | 845 | May 2006 |
| Fajã da Nogueira – Til Gigante | 32.7457 | -16.9150 | 841 | May 2006 |
| Fanal – Recta do Sargento caminho | 32.8236 | -17.1560 | 890 | May 2006 |
| Fanal – Recta do Sargento largo | 32.8226 | -17.1539 | 889 | May 2006 |
| Fanal – Rile Amarelo | 32.8182 | -17.1521 | 1023 | May 2006 |
| Funduras – Casa Abrigo | 32.7494 | -16.7917 | 561 | May 2011 |
| Funduras – norte | 32.7540 | -16.8099 | 552 | May 2006 |
| Funduras – sul | 32.7493 | -16.8114 | 500 | May 2006 |
| Galhano Baixo | 32.8017 | -17.1669 | 697 | July 2012 |
| Galhano Médio | 32.8003 | -17.1708 | 873 | July 2012 |
| Galhano Topo | 32.7967 | -17.1750 | 952 | July 2012 |
| Ginjas | 32.7758 | -17.0534 | 869 | June 2007 |
| Levada da Serra do Faial | 32.7372 | -16.8483 | 860 | June 2011 |
| Levada dos Cedros | 32.8259 | -17.1580 | 820 | May 2006 |
| Levada Ponte Roquete | 32.7391 | -16.9156 | 1074 | May 2006 |
| Miradouro das Voltas | 32.8077 | -16.9499 | 848 | June 2011 |
| Montado do Leacoque – norte | 32.7418 | -16.9177 | 614 | May 2006 |
| Montado do Leacoque – sul | 32.7415 | -16.9161 | 630 | May 2006 |
| Montado dos Pessegueiros Baixo | 32.8090 | -17.0714 | 462 | July 2012 |
| Montado dos Pessegueiros Médio | 32.7958 | -17.0853 | 1284 | July 2012 |
| Montado dos Pessegueiros Topo | 32.7944 | -17.0866 | 1338 | July 2012 |
| Pico das Pedras Baixo | 32.7841 | -16.9055 | 883 | May 2006 |
| Pico das Pedras Topo | 32.7689 | -16.9117 | 1254 | May 2011 |
| Posto do Cascalho | 32.8041 | -16.9466 | 827 | June 2011 |
| Queimadas | 32.7873 | -16.9047 | 841 | May 2006 |
| Rabaçal | 32.7647 | -17.1341 | 930 | June 2007 |
| Ribeira da Cruz | 32.8262 | -17.2098 | 768 | May 2011 |
| Ribeira da Janela | 32.8345 | -17.1785 | 402 | July 2012 |
| Ribeira do Tristão | 32.8407 | -17.1912 | 717 | May 2011 |
| Ribeiro Bonito – Levada | 32.8047 | -16.9346 | 568 | May 2006 |
| Ribeiro Bonito – Ribeiro | 32.7985 | -16.9360 | 560 | May 2006 |
| Ribeiro Frio – Cottages | 32.7319 | -16.8861 | 994 | May 2006 |
| Ribeiro Frio – Viveiro | 32.7354 | -16.8864 | 906 | May 2006 |
| Risco | 32.7608 | -17.1256 | 1048 | June 2007 |
| Tornos – este | 32.7715 | -16.9655 | 664 | July 2012 |
